# A Case of Severe Bronchial Asthma Controlled with Tacrolimus

**DOI:** 10.1155/2011/479129

**Published:** 2011-04-06

**Authors:** Hirokazu Taniguchi, Kotaro Tokui, Yasuhiro Iwata, Hitoshi Abo, Saburo Izumi

**Affiliations:** ^1^The Department of Internal Medicine, Toyama Prefectural Central Hospital, 2-2-78 Nishinage, Toyama 930-8550, Japan; ^2^The Department of Radioloy, Toyama Prefectural Central Hospital, 2-2-78 Nishinage, Toyama 930-8550, Japan

## Abstract

*Background*. The control of severe bronchial asthma, such as corticosteroid-resistant asthma, is difficult. It is also possible that immunosuppressive agents would be effective for bronchial asthma. *Case Summary*. A 55-year-old Japanese female presented with severe bronchial asthma controlled with tacrolimus. She had been diagnosed with bronchial asthma during childhood. Her asthma worsened, and a chest radiograph showed atelectasis of the left lung. Bronchoscopy revealed the left main bronchus to be obstructed with viscous sputum consisting of 82% neutrophils and no eosinophils. The atelectasis did not improve with corticosteroid treatment, but was ameliorated by administration of tacrolimus. *Discussion*. This patient had severe asthma due to neutrophilic inflammation of the airways. Tacrolimus is effective for treating severe asthma, for example, in corticosteroid-resistant cases.

## 1. Introduction

The control of severe bronchial asthma, such as corticosteroid-resistant asthma, is difficult [1]. T cells are strongly associated with bronchial asthma [2]. It has also been suggested that immunosuppressive agents suppressing T cells would be effective for the treatment of bronchial asthma [3]. Immunosuppressive agents have recently been administered in rare cases of severe bronchial asthma. 

 This case report describes a patient suffering from severe bronchial asthma, associated with airway inflammation due to neutrophil infiltration, which could be controlled by the administration of tacrolimus.

## 2. Clinical Summary

**Figure 1:**
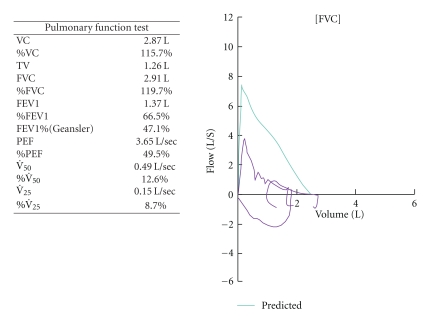
The results of the pulmonary function tests three months before admission.

A 55-year-old Japanese female was admitted with infected traumatic cutaneous ulcers. The infectious organism was *Staphylococcus aureus*, and levofloxacin was administered. She had been diagnosed with bronchial asthma during childhood and had been treated by her family doctor. For several years, her bronchial asthma worsened and she was treated with fluticasone/salmeterol (500 mcg/100 mcg per day), prednisolone (10 mg per day), theophylline (400 mg per day), and pranlukast (leukotriene receptor antagonist, 450 mg per day). She had suffered from dyspnea upon exertion and wheezing continuously for the prior two months. Pulmonary function tests, which had been conducted three months before admission, showed a pattern of obstruction (Figure 1). 

**Figure 2:**
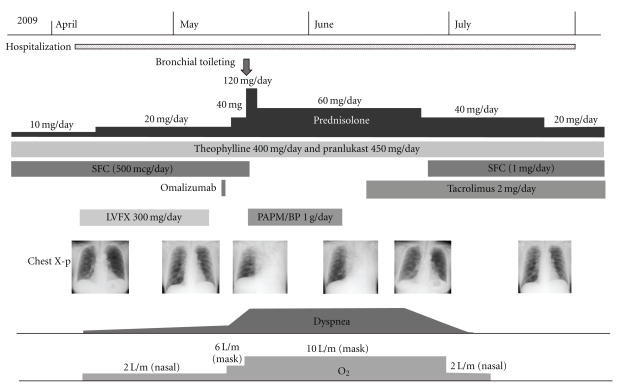
The clinical course. (SFC: fluticasone/salmeterol, LVFX: levofloxacin, and PAPM/BP: panipenem/betamipron.)

**Figure 3:**
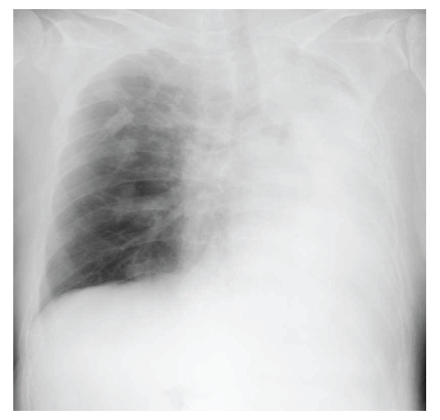
A chest radiograph showed atelectasis of the left lung.

**Figure 4:**
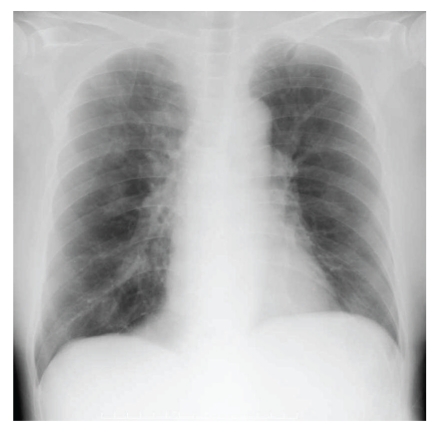
A chest radiograph showed an improvement in the atelectasis of the left lung.

 Her bronchial asthma showed exacerbation after admission. Laboratory studies revealed a serum lactate dehydrogenase (LDH) level of 287 international units (IU) per liter, myeloperoxidase-antineutrophil cytoplasmic antibody (MPO-ANCA) levels less than 1.3 IU per milliliter, and an immunoglobulin E (IgE) level of 635 IU per milliliter; no peripheral blood eosinophils were detected (Table 1). Her prednisolone dose was increased to 20 mg per day, but she developed dyspnea, wheezing, and hypoxia necessitating inhalation of oxygen. This exacerbation of her bronchial asthma persisted for a month, despite the increase in prednisolone. Next, 300 mg per day of omalizumab was administered; however, her dyspnea persisted (Figure 2) and her chest radiograph showed atelectasis of the left lung (Figure 3). Her CRP level was 0.27 mg per liter. Bronchoscopy revealed that the left main bronchus and other left bronchi were obstructed with colorless viscous sputum. Due to the large volume of viscous sputum in the peripheral bronchi, bronchial toileting did not improve the atelectasis. No eosinophils or malignant cells were identified in suction specimens of the sputum, which contained 82% neutrophils and 18% lymphocytes, and no bacteria or fungi were detected by sputum cultures. The prednisolone dose was increased to 120 mg per day for three days and then to 60 mg per day for the remainder of the hospitalization. Panipenem/betamipron (carbapenem antibiotic) was administered concurrently with the prednisolone treatment for two weeks. However, the patient continued to demonstrate severe dyspnea, wheezing, and hypoxia that required the inhalation of a large amount of oxygen, and her atelectasis did not improve. One month later, she was treated with 2 mg per day of tacrolimus. Her symptoms and respiratory status did not change during the first week, but, thereafter, the atelectasis improved (Figure 4). Thereafter, her dyspnea and hypoxia started to gradually improve. After three months of tacrolimus administration, oxygen administration was stopped and the patient became capable of short walks. The prednisolone was tapered over the course of four weeks to 20 mg per day, and she was transferred to another hospital for rehabilitation.

## 3. Discussion

In the current patient, we suspected that the cause of atelectasis was exacerbation of bronchial asthma. This case was not considered to have allergic bronchopulmonary aspergillosis based on negative findings for IgE specific to aspergillus, negative suction sputum cultures, no peripheral blood eosinophils, and no central bronchiectasis. We also ruled out the possibility of allergic bronchopulmonary candidiasis, because the only positive finding was IgE specific to candida. In addition, there were no signs of bacterial infection in her lungs, as demonstrated by negative serum CRP, negative suction sputum specimen cultures, and a lack of response to antibiotic treatment.

 The basic pathology of bronchial asthma is chronic airway inflammation with infiltration of inflammatory cells such as eosinophils. Asthmatics with severe disease have predominantly neutrophilic inflammation, in contrast to asthmatics whose disease is controlled [4, 5]. The significance of neutrophilic inflammation is not apparent, but when such inflammation is not controlled by corticosteroid therapy, there is an association with increased severity in a subset of severe asthma cases. The current patient's suction sputum specimens, which were obtained during bronchoscopy, included many neutrophils but no eosinophils. Therefore, the patient demonstrated severe asthma due to neutrophilic inflammation.

 Tacrolimus is an immunosuppressive agent. This drug has a mode of action which is similar to that of cyclosporine, namely due to its ability to inhibit calcineurin in T cells. Some previous studies on humans regarding steroid-dependent asthma treated with oral cyclosporine have been reported [6, 7]. These results, therefore, suggest that cyclosporin might be beneficial in some patients with steroid-dependent asthma, while not being of any benefit in other patients.

 Tacrolimus suppresses the activation of T cells [8] and the production of interleukin (IL)-2, IL-3, IL-4, IL-5, IL-6 [9], granulocyte/macrophage colony-stimulating factor (GM-CSF)  [10], interferon-gamma, and other proteins. Tacrolimus is thought to control severe asthma by suppressing the activation of T cells [2]. In this case, severe asthma was not controlled only with tacrolimus but also by many other treatments administered prior to tacrolimus. Tacrolimus would have provided additional benefits. Helper T lymphocytes play a very important role in the development and progression of asthma [2], and these T lymphocytes probably played a similar role in this case. In our patient, tacrolimus may have controlled severe asthma by suppressing T cell activation [2], which would result in the suppression of airway inflammation. It is followed by that narrowing of the airways, hypersecretion of inspissated mucus, as well as airway mucosal capacity for excretion of mucus, all improved. As a result, the viscous sputum in the left bronchus and other left bronchi disappeared and atelectasis thereby improved. However, neutrophils were the predominant cell type associated with airway inflammation in our patient. Neutrophils are activated by multiple factors, including IL-6, IL-8, leukotriene B4, and GM-CSF [11]. It may also be that tacrolimus controls severe asthma by suppressing the activation of neutrophils via the suppression of IL-6 expression or by some other as yet unknown mechanisms. GM-CSF suppresses the activation of neutrophils, but strongly activates eosinophils. Moreover, it is interesting that corticosteroid therapy prolongs neutrophil survival and accelerates neutrophilic airway inflammation by preventing apoptosis [12].

 We have presented a case of severe bronchial asthma, associated with airway inflammation characterized by neutrophil infiltration, controlled with tacrolimus. Therefore, this drug should be considered for the treatment of severe asthma, including cases demonstrating corticosteroid resistance.

##  Conflict of Interests

The authors report that there are no significant conflict of interests regarding any companies/organizations whose products or services may be discussed in this paper.

## Figures and Tables

**Table 1:** Laboratory data obtained at the start of the exacerbation of bronchial asthma. (UniCAP specific IgE: Fluoroenzymeimmunoanalyses specific immunoglobulin E.)

Hematology	
White blood cells	14,500/mm^3^
Neutrophils	91.0%
Eosinophils	0.0%
Lymphocytes	6.0%
Monocytes	3.0%
Basophils	0.0%
Red blood cells	396 × 10^4^/mm^3^
Hemoglobin	11.5 g/dl
Hematocrit	33.9%
Platelets	25.1 × 10^4^/mm^3^
Biochemistry	
Total protein	5.1 g/dl
Albumin	3.3 g/dl
Lactate dehydrogenase	287 IU/l
Aspartate aminotransferase	10 IU/l
Alanine aminotransferase	16 IU/l
Creatin phosphokinase	21 IU/l
Blood urea nitrogen	6 mg/dl
Creatinine	0.3 mg/dl
Fasting blood suger	116 mg/dl
Serology	
C-reactive protein	0.28 mg/dl
Myeloperoxidase-antineutrophil cytoplasmic autoantibodies	<10 EU
Antinuclear antibodies	negative
Immunoglobulin E	635 IU/ml
Aspergillus antigen	(−)
Candida antigen	(−)
UniCAP specific Immunoglobulin E	
*Dermatophagoides farinae *	<0.34 UA/ml
*Dermatophagoides farinae *	<0.34 UA/ml
House dust 1	<0.34 UA/ml
House dust 2	0.45 UA/ml
Aspergillus	<0.34 UA/ml
Candida	22.30 UA/ml
